# Fabrication of Chitosan Silk-based Tracheal Scaffold Using Freeze-Casting Method

**DOI:** 10.18869/acadpub.ibj.21.4.228

**Published:** 2017-07

**Authors:** Zeinab Nematollahi, Mohammad Tafazzoli-Shadpour, Ali Zamanian, Amir Seyedsalehi, Shadmehr Mohammad-Behgam, Fariba Ghorbani, Fereshte Mirahmadi

**Affiliations:** 1 Faculty of Biomedical Engineering, Amirkabir University of Technology, Tehran, Iran; 2 Materials and Energy Research Center, Karaj, Iran; 3 Tracheal Diseases Research Center, National Research Institute of Tuberculosis and Lung Diseases (NRITLD), Shahid Beheshti University of Medical Sciences, Tehran, Iran

**Keywords:** Chitosan, Trachea, Therapeutic substitution, Regeneration, Tissue scaffold

## Abstract

**Background:**

Since the treatments of long tracheal lesions are associated with some limitations, tissue engineered trachea is considered as an alternative option. This study aimed at preparing a composite scaffold, based on natural and synthetic materials for tracheal tissue engineering.

**Methods:**

Nine chitosan silk-based scaffolds were fabricated using three freezing rates (0.5, 1, and 2°C/min) and glutaraldehyde (GA) concentrations (0, 0.4, and 0.8 wt%). Samples were characterized, and scaffolds having mechanical properties compatible with those of human trachea and proper biodegradability were selected for chondrocyte cell seeding and subsequent biological assessments.

**Results:**

The pore sizes were highly influenced by the freezing rate and varied from 135.3×372.1 to 37.8×83.4 µm. Swelling and biodegradability behaviors were more affected by GA rather than freezing rate. Tensile strength raised from 120 kPa to 350 kPa by an increment of freezing rate and GA concentration. In addition, marked stiffening was demonstrated by increasing elastic modulus from 1.5 MPa to 12.2 MPa. Samples having 1 and 2°C/min of freezing rate and 0.8 wt% GA concentration made a non-toxic, porous structure with tensile strength and elastic modulus in the range of human trachea, facilitating the chondrocyte proliferation. The results of 21-day cell culture indicated that glycosaminoglycans content was significantly higher for the rate of 2°C/min (12.04 µg/min) rather than the other (9.6 µg/min).

**Conclusion:**

A homogenous porous structure was created by freeze drying. This allows the fabrication of a chitosan silk scaffold cross-linked by GA for cartilage tissue regeneration with application in tracheal regeneration.

## INTRODUCTION

The idea of using an alternative for the trachea is corresponding to the Belsey’s idea about infeasibility of tracheal resection exceeded than two centimeters[1]. However, other researchers could go beyond this limit[2]. Now, resection and a safe anastomosis of one-half of the tracheal lesion in adults and one-third in children are feasible. Though, for long segments, tracheal substitutes are mandatory[3]. In this regard, a wide range of options is available including autologous tissue flaps and patches, synthetic stents and prostheses, and tissue-engineered scaffolds[3]. However, attempts to reconstruct human trachea have been relatively unsuccessful due to the mechanical complexity of the tissue and exposing to the non-sterile environment[4].

Similar to other artificial organs, tracheal replacement necessitates the foreign materials to be biocompatible, nontoxic, nonimmunogenic, non-carcinogenic and facilitate epithelial lining[3,5,6]. In practice, the field of trachea tissue engineering has experienced different approaches for preparing an apt tracheal alternative over the time. In this regard, both natural and biological scaffolds have been employed with various results. Several synthetic biomaterials have been used as tracheal scaffolds such as polyester polymers, stainless steel, silicon, teflon, vitallium, glass, and polyethylene[7,8]. However, natural biomaterials are usually used because of their well-established biocompatiblity and biofuctional properties[9]. Biomaterials such as silk and chitosan[10], collagen[11], and hyaluronic acid[12] have been candidates for trachea tissue engineering. Conversely, inadequate mechanical properties of natural materials[13], as well as the high rate of biodegradability[14] have limited their applications. Despite initial successes, the first artificial trachea based on the synthetic scaffold led to the generation of granulation tissue due to poor biocompatibility[15]. Hence, the fabrication of composites through the combination of natural and synthetic components, or the enhancement of structural properties of natural materials through novel fabrication techniques have been the major strategies in trachea tissue engineering[16].

Chitosan is a biocompatible and biodegradable natural-derived polymer used as a biomaterial because of its non-toxic and low-immunogenicity properties, antibacterial activity, and wound-healing properties[17]. Chitosan is structurally similar to glycosaminoglycans (GAGs) that exist in cartilage; hence, it has been suggested as a proper base for cartilage tissue engineering[18]. Due to inadequate mechanical properties of chitosan that provide limited applications as scaffolds, the combination of chitosan with other natural or synthetic polymers is well proposed. Among natural polymers, silk fibroin has been used in tissue engineered scaffolds[19] owing to its biocompatibility, degradation kinetics, and regenerative capacity. To date, silk fibroin-chitosan scaffold has been successfully employed for abdominal wall reconstruction, skin wound healing, and the support of bone regeneration, which indicate favorable handling characteristics and dynamic local tissue integration properties[17,20]. Previous research on chitosan silk scaffolds has also shown promising results in providing a biocompatible substitute for reconstruction of small defects in cartilage[21]; however, these biomaterials have not been well applicable in trachea replacement. The convenient chitosan silk biomaterials either in hydrogel form or solid may not afford to provide enough mechanical performance and appropriate degradability. Therefore, alternative methods of fabrication together with strong crosslinkage may be appropriate strategies for trachea engineering.

Freeze casting, known as ice templating, which has gained a great deal of attention over the past few years, is a versatile technique for producing porous materials including selective polymers. It has also been developed to tailor the pore structure of porous materials by manipulating the degree of porosity, pore size, pore shape, and pore orientation[22,23]. This method have shown many benefits, such as the adaptability of the process as well as providing high strength and a variety of pore sizes, and environmental friendliness[24]. Hence, in addition to being economical in mass production, such method may be a candidate for fabrication techniques of chitosan silk scaffolds.

In this study, we developed chitosan silk-based scaffolds through the freeze-casting technique, and used glutaraldehyde (GA) as the crosslinking agent to enhance biocompatibility and mechanical strength in tissue engineering of the trachea, through the adjustment of different freeze rates and the amount of the crosslinking agent.

## MATERIALS AND METHODS

Silk fibroin was extracted from silkworm cocoons, dissolved in triple solution (ethanol-water-CaCl_2_) and mixed with chitosan solution with a ratio of 3 to 1. The resultant solution was freeze-casted with freezing rates of 0.5, 1, and 2ºC/min and GA amounts of 0, 0.4, and 0.8 wt%. Samples were then freeze-dried, and the obtained nine scaffolds were prepared for further experiments. Prepared scaffolds were characterized by scanning electron microscopy (SEM) and Fourier transform infrared spectroscopy (FTIR). Mechanical and swelling properties, the size and distribution of porosity, biodegradability, and biocompatibility were assessed as well. The scaffolds having suitable mechanical properties compatible with the human trachea and proper biodegradability were selected for cell seeding and biological analyses.

### Preparation of silk fibroin

Bombyx mori silkworm cocoons were obtained from Gilan University (Rasht, Iran). Raw silk at 1:100 (w/v) was added to 0.02 M Na_2_CO_3_ solution and was boiled for 1 h to remove sericin. Subsequently, raw silk was drained and heated in distilled water at 100°C for 30 min and finally rinsed in running distilled water to remove any remaining sericin. After drying, the degummed silk was dissolved in water/ethanol/calcium chloride (molar weight ratio 8:2:1) solution at 80°C for about 40 min. The solution was then dialyzed (MWCO 12000 Da, Sigma. USA) against deionized water for three days, with changing water every day, to remove the salt[25].

### Fabrication of the scaffold

Medium-molecular-weight chitosan (Sigma-Aldrich, USA) was dissolved in 2% (w/v) acetic acid solution. As previously suggested, the 75/25 ratio of silk to chitosan gives adequate mechanical properties compared to other ratios[17]. Hence, 7.5% (w/v) silk solution and 2.5% (w/v) chitosan solution were blended at 50:50 (v/v), and the cross-linking agent of 0, 0.4, and 0.8 wt% GA (Sigma-Aldrich, Gillingham, England) was added to the solution. The scaffolds were prepared by the freeze-casting method that enables the control of porous structure through different freezing rates and provides higher mechanical properties in freezing direction. Freeze casting was performed by pouring the blended solution into a polytetra-fluoroethylene mold mounted on a copper cylinder. The mold temperature was controlled by adding liquid nitrogen and a ring heater connected to the copper cylinder and proportional integral derivative controller. The temperature was monitored by a thermocouple located near the surface of the cold cylinder during fabrication. Three different freezing rates were utilized as 0.5, 1, and 2°C/min. After removing from the mold, the frozen samples were dried in a freeze dryer (Beta 1-2LD plus; Martin Christ GmbH, Osterode, Germany) at a lower temperature of -55°C and the pressure of 2.1 Pa for 72 h. The samples were named as RxGy, in which R and G stood for freezing rate, glutharaldehyde. The values of R and G were denoted as x and y, respectively. Table 1 shows samples specifications.

**Table 1:** Specifications of sample based on different glutaraldehyde (GA) concentration and freezing rate

Process parameters	R0.5G0	R0.5G0.4	R0.5G0.8	R1G0	R1G0.4	R1G0.8	R2G0	R2G0.4	R2G0.8
Freezing rate (°C/min)	0.5	0.5	0.5	1	1.0	1.0	2	2.0	2.0
GA (wt%)	0	0.4	0.8	0	0.4	0.8	0	0.4	0.8

### Scaffold characterization

### FTIR spectroscopy

To ensure the purity and accuracy of extracted silk fibrin solution, the infrared spectra of the dried samples in KBr tablets were assessed using a FTIR spectrophotometer (Perkin Elmer Spectrum 400, Germany) in the range of 450-3950 cm^-1^.

### SEM analysis

The dried samples were cut with a sharp blade to expose internal microstructure. The morphology and microstructure of the scaffolds were studied with a scanning electron microscope (Stereo scan S 360-Leica, Cambridge, England). Because of the poor conductivity of the samples, a thin layer of gold was coated on the surface of the scaffold before testing. Pore sizes were determined in both perpendicular and parallel axes to the freezing direction by KLONK Image Measurement software 14.1.2. At least 20 measurements were conducted for each sample.

### Analysis of mechanical properties

Cylinder-shaped samples were cut into rectangular strips[26] of 20×10×4 mm and were subjected to tensile test using Instron 5565 (Instron Corp., USA) with the cross head speed of 0.5 mm/min. Each test was repeated five times, and the average and the standard deviation of related parameters were determined. The force-displacement data obtained from tests were converted to stress-strain data considering the dimensions of the samples, and the slope of the curve fitted to the stress-strain data was measured as the Young’s modulus of elasticity. Furthermore, the stress at which the samples failed was calculated as the strength of scaffold from the failure load in a tensile test.

### PBS uptake assessment

For determination of PBS uptake (swelling ration), three samples of each scaffold were prepared, and their dried weight (W_d_) was measured. Samples were then submerged in PBS (pH 7.4) at 37°C for 30 min. After drying the surface of samples with filter paper, the swollen weight (W_s_) was measured. The equilibrium water uptake was determined by PBS adsorption percentage as follow:


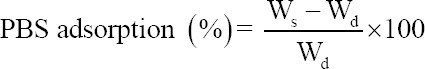


### Degradability analysis

To examine the degradation rate of the scaffolds, the dried weight of each sample was measured at the beginning and on days 7, 15, 35, 55, and 70[27]. The samples were submerged into the PBS solution and put inside a thermo-shaker. The PBS solution was replaced every day. The samples were dehydrated at 40°C for 12 h, and then the dried sample weights were recorded on the specific days. The degradation percentage of each sample was calculated.

### *In vitro* cell culture and biocompatibility analysis

The optimized samples for tracheal tissue engineering were selected for *in vitro* cell culture based on the best results obtained for mechanical characteristics, porosity, and biodegradability. MTT assay, cell adherence, and GAG content were compared with the cell culture of allogeneic primary chondrocytes.

### Chondrocytes isolation

Articular chondrocytes were isolated from knee joints of New Zealand white rabbits. All experiments were in compliance with the Ethical Committee of National Cell Bank of Pasteur Institute of Iran (Tehran). The cartilaginous tissues were cut off from the joint, washed several times with antibiotic containing medium and then minced to small pieces. The obtained pieces were predigested in 0.25% Trypsin-EDTA solution (Sigma-Aldrich, USA) for 1 h and then digested in the collagenase type II solution (0.08 mg/mL, Sigma-Aldrich, USA) for 12 h inside an incubator. The resulting cell suspension was filtered through a 40-μm filter and then centrifuged at 60 ×g for 5 min and plated in DMEM (GIBCO, Scotland)/Ham’s F12 supplemented with 10% FBS (Seromed, Germany) inside the incubator at 37°C, 5% CO_2_. The medium was changed every three days. The second passage cells were used for cell seeding on scaffolds.

### Cell culture

For *in vitro* cell culture, the size of 1×1 cm of scaffolds was sterilized and placed in 6-well plates. A total number of 5×10^5^ cells/ml from passage 2 of chondrocytes were trypsinized and cultured on R1G0.8, R2G0.8, and on the plate as control group. The culture medium included 500 µl of DMEM (GIBCO, Scotland)/Ham’s F12 culture medium solution supplemented with 10% (v/v) FBS (Seromed, Germany), 100 U/ml penicillin, and 100 μg/ml streptomycin exchanged every three days. After 14 and 21 days of cell culture, scaffolds containing chondrocytes as well as the control group were evaluated for cell adhesion and GAG content.

### MTT assay

Specimens with the dimensions of 1×1 cm were sterilized by submerging in ethanol 100%, ethanol 70%, and ethanol 50% for 1 hour, 30 min, and 30 min, respectively, followed by washing with sterilized PBS[28]. The sterile scaffolds (3±0.5 cm^2^/mL of culture medium) were placed in a 12-well tissue culture plate, washed three times with fresh culture medium and incubated in fresh culture medium at 37°C for 1, 3, and 14 days to get scaffold extract. As the control group, serum-supplemented culture medium (DMED) at the same condition was used. Then the cells were seeded into 96-well plates at a density of 1×10^4^ cells/mL. The culture medium (100 μL) of cells in 96-plates was replaced with obtained extracts as so the control group medium. After 24 h of incubation, the viability of each group was assessed using MTT (Sigma-Aldrich, USA). The medium was removed, and 100 μL of a 0.5 mg/mL MTT solution was added to each well and incubated at 37°C for 4 h. Finally, MTT solution was removed gently and 150 μL isopropanol (Sigma-Aldrich, USA) was added to each well to dissolve the MTT formazan purple crystals. The absorbance of the solutions was measured at 545 nm using an ELISA Reader (Stat Fax-2100, USA). The normalized relative viability or cell growth (%) was calculated from the following equation:


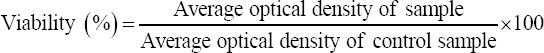


### Determination of glycosaminoglycans

GAG content was determined using dimethylmethylene blue (DMMB, Sigma-Aldrich USA) test [29]. A total of 5×10^3^ cells were cultivated on selected scaffolds with the size of 0.5×0.5 cm. After 14 and 21 days, while half of the medium was replaced each three days, 500 μL of medium was taken out for further evaluation. Then 1.5 mL acetone (Merck, Germany) was added to the collected medium and kept at -20°C for 24 h. Samples were centrifuged at 260 ×g at 4°C for 30 min. The precipitated material was suspended in 100 μL PBS containing papain (20 μg/mL), activated with 5 mM cysteine, and followed by incubation at 60°C for 16 h and boiling for 15 min. A working standard solution of chondroitin sulfate C (shark cartilage extract, Sigma-Aldrich, USA) was prepared. The DMMB assays were performed in 96-well plates using an ELISA plate reader and the optical density was immediately measured at 545 nm.

### Cell adhesion evaluation

To evaluate cell attachment, cell-seeded scaffolds were fixed with 2.5% GA (Merck, Germany) at room temperature for 2 h, rinsed in distilled water and dehydrated through alcohol gradient. Samples were dried naturally for analysis by SEM (AIS2100, South Korea).

### Statistical analysis

Sample values were expressed as means±standard deviation (SD). One-way ANOVA on Ranks, Tukey’s test post hoc between all groups, or *t*-test between two groups at α=0.05 was carried out to assess the significant effect of process factors on different biological and non-biological parameters.

## RESULTS

### FTIR spectroscopy

**Fig. 1:**
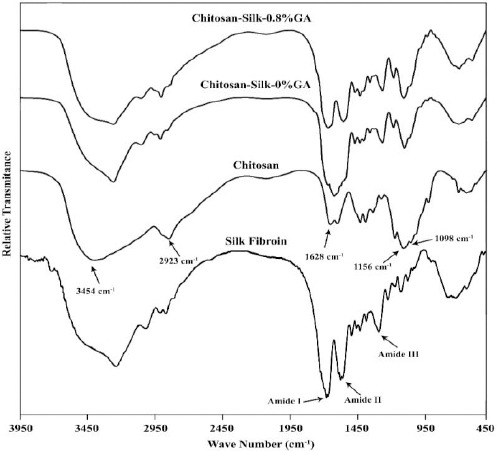
Fourier transform infrared spectroscopy of pure chitosan and chitosan silk composites before and after crosslinking with 0.8 wt% weight of glutaraldehyde. Waves show the presence of both chitosan and silk in the composite structure and the effect of glutaraldehyde (GA) to form new bands.

The results of FTIR analysis were consistent with published data and denoted the presence of both chitosan and silk in the composite structure and also confirmed the effect of GA to form new bands (Fig. 1). The results of the pure chitosan showed strong absorption bands of -OH and N-H at 3454 cm^-1^. The peak for asymmetric stretch of C-O-C was found at around 1150 cm^-1^[30]. The peak at about 2923 cm^-1^ indicated the asymmetrical stretching vibrational mode for -CH_2_ group[31]. Moreover, peaks at 1628 and 1156 cm^-1^ were related to the stretching mode of C=O and sulfate group of polysaccharides, respectively[32]. Lastly, the band at 1098 cm^-1^ corresponded to the stretching vibration of the C-O bond in chitosan[19].

Protein substances were characterized by amide I, amide II, and amide III bands that could be seen at 1580-1700, 1520-1565, and 1200-1300 ranges, respectively[33]. As shown in Figure 1, the peaks at about 1646 (C=O stretching), 1537 (N-H bending), and 1236 cm^-1^ (C-N stretching) could be attributed to amide bands of silk.

FTIR spectra of the composition of chitosan and silk-fibroin showed specific bonds at 3435 cm^-1^, which were related to -OH and N-H. There were also other peaks corresponding to chitosan, including -CH_2_ at 2927 cm^-1^, stretching mode of C=O at 1598 cm^-1^, and bending bond of N-H at 680 cm^-1^. In addition, the peak at 1230 cm^-1^ showed the silk fibroin in the scaffold. On the other hand, the FTIR spectra of cross-linked scaffolds indicated shifting in a peak at 1628 cm^-1^ to 1664 cm^-1^ due to the formation of C≡N group. There was also another peak corresponded to the symmetric stretching bond of C-O-C.

The amide peaks were common between all synthetic, natural or composite polymeric materials that had peptide (CO-NH) bond in their structure and were appeared in well-known ranges. Chitosan is a partially deacetylated chitin in which acetyl group is exposed on some repeated units via peptide bond. Moreover, vibrational peak of primary amine groups (-NH_2_), which were formed on chitosan backbone through deacetylation processing, was located at amide I band. Therefore, FTIR spectrum of chitosan overlapped with silk, and the characteristic peaks of silk at their specific wave numbers were combined with chitosan amide bands or shifted to another wave number.

### SEM and porosity measurement

**Fig. 2:**
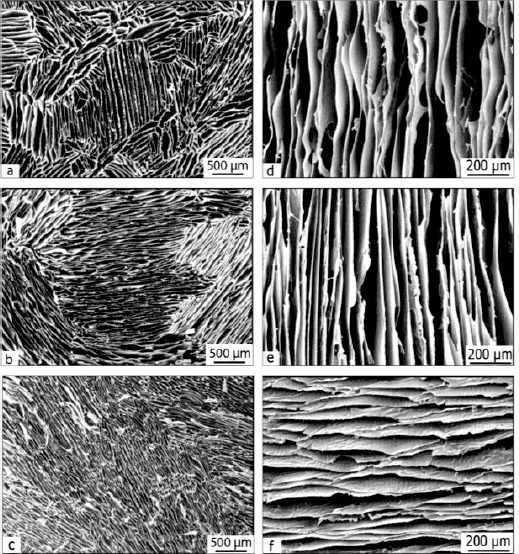
Scanning electron micrograph of the scaffolds in both parallel and perpendicular to solidification direction. (a) R0.5G0.8, perpendicular; (b) R1G0.8, perpendicular; (c) R2G0.8 perpendicular, (d) R0.5G0.8 parallel; (e) R1G0.8 parallel; (f) R2G0.8, parallel. The uniform morphology of the pores is created by constant freeze-casting rate at -196ºC.

**Fig. 3:**
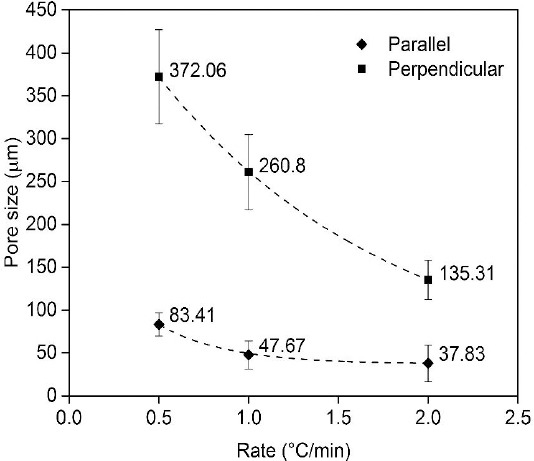
Pore sizes of fabricated scaffolds due to different rates based on 0.8 wt% glutaraldehyde content. The higher freezing rates correspond to the smaller pore sizes.

The porous microstructure and morphology of the chitosan silk scaffolds were studied by SEM in both perpendicular and parallel directions of solidification (Figs. 2 and 3). Despite a decrease in pore size, the higher freezing rate caused more ice nucleation, which resulted in higher number of pores. Based on SEM morphological analysis using KLONK Image Measurement 14.1.2, minimum, and maximum pore sizes were 135.3 and 372.1 μm in a perpendicular direction and 37.8 and 83.4 μm in vertical direction, respectively. Despite noticeable differences in pore size among the samples, all pore sizes were considered to be appropriate for chondrocyte in growth[34]. Consequently, increasing the freezing rate resulted in pore size decrement did not cause limitations for cell growth. There were significant differences in pore size between R1 and R2 scaffolds (*P*<0.05), and R0.5 had also smaller pore size compared to R1 (*P*< 0.05).

### Mechanical properties

**Fig. 4:**
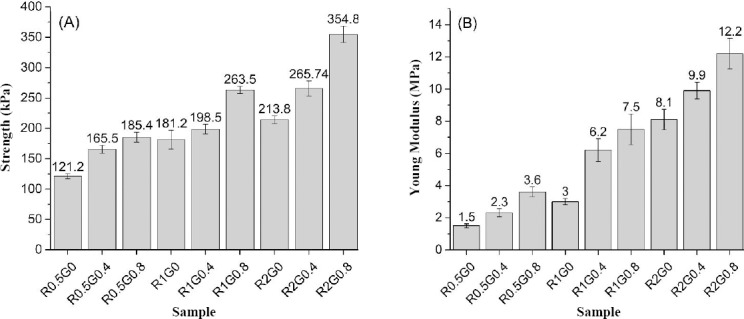
Mechanical properties. (A) Scaffolds tensile strength (B) Young’s modulus (n=3). Tensile strength and elastic modulus were under the influence of the both glutaraldehyde content and freezing rate.

Figure 4 shows the results of the tensile strength and Young’s modulus of elasticity. Both GA treatment and increasing the freezing rate resulted in the elevation of the tensile strength of the scaffolds from 120 kPa to about 350 kPa and marked stiffening through increasing elastic modulus from 1.5 MPa to 12.2 MPa. There were no significant differences among the strength value of R0.5G0.4 vs. R0.5G0.8, R1G0.4 vs. R1G0, R1G0 vs. R2G0, R1G0.8 vs. R2G0.4, and R2G0.4 vs. R2G0 (*P*>0.05). R0.5G0 had a tensile strength of 121.2±10.3 kPa, which was significantly lower than all other scaffolds. Additionally, R2G0.8 strength (354.8±23.7 kPa) had the highest score among all samples (*P*<0.05).

Obtained data indicated that both GA concentration and the freezing rate had a remarkable effect on R0.5G0 but when the rate was 0.5 or 1°C/min, an increase in crosslinking agent from 0.4 to 0.8 wt% did not play a notable role in the strength value. In the absence of GA, an increase in the freezing rate from 1 up to 2°C/min did not affect scaffolds strength (*P*>0.05). The additional increase in GA from 0.4 to 0.8 wt% and a decrease in the freezing rate from 2 to 1 °C/min had an equal effect on the tensile strength of the samples.

Young’s modulus of each scaffold was significantly different in comparison with other ones (*P*<0.05). Sample R0.5G0 had the lowest Young’s modulus (1.5±0.02 MPa) and R2G0.8 with the modulus of 12.2±2.4 MPa had the highest value. In comparison with strength value, any changes in GA concentration made a significant difference in the Young’s modulus. On the other hand, the increment of GA from 0.4 to 0.8 wt% had a significant effect on modulus. Also, in the absence of GA, an increase in the freezing rate from 1 up to 2°C/min strongly affected scaffolds in term of Young’s modulus.

### PBS uptake

**Fig. 5:**
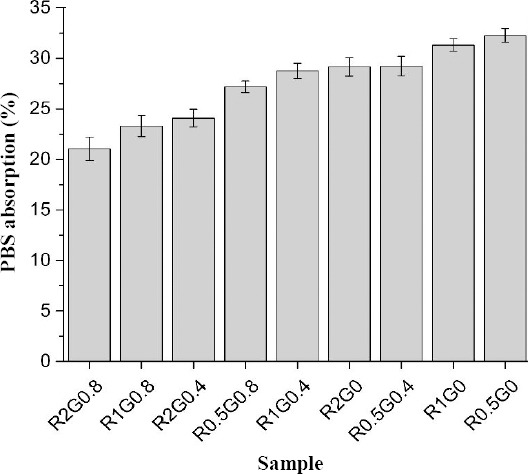
PBS uptake in all the prepared scaffolds. Values are mean±S.D. (n=3). Swelling ratio was decreased by increasing glutaraldehyde content and freezing rate.

Figure 5 shows the results of PBS uptake (weight of PBS/dry sample weight) by samples at room temperature. It was observed that both GA concentration and freezing rate had negative effects on the PBS absorption, and swelling ratio was decreased by increasing GA concentration. Samples R2G0.8, R1G0.8, and R2G0.4 had swelling ratios of 21.02±2.08, 23.28±2.91, and 24.08±3.65, respectively, which were statistically lower than those of all the other scaffolds (*P*<0.05). In addition, R0.5G0 and R1G0 composites had statistically higher swelling ratio (32.24 ±3.59, 31.30±3.06, respectively) than either R2G0 (29.15±2.07) or R2G0.4 (24.08±3.46) (*P*<0.05). There was no statistical difference between R1G0.4 and R0.5G0.8 (*P*>0.05). The minimum value of PBS uptake was related to the samples with 2°C/min freezing rate and 0.8 wt% GA (21%), and the maximum absorption belonged to the ones with 0.5°C/min rate without crosslinking agent (32%).

### Degradability analysis

Table 2 shows the result of degradability analysis on days 7, 15, 35, 53, and 70. Results indicated that both GA value and freezing rate affected degradation rate. In the absence of GA, samples dissolved thoroughly within 15 days. Higher GA values caused lower degradation rate most probably due to the higher number of bonds. As shown in Figure 3, an increase in freezing rate led to decreasing the pore size, which in turn caused lower PBS absorption, thereby lowering degradation rate.

**Table 2:** Degradibility percentange of the scaffolds

Sample	Day 7	Day 15	Day 35	Day 53	Day 70
R0.5G0	35.66635	Fail	Fail	Fail	Fail
R0.5G0.4	6.2954	27.23971	43.94673	62.46973	Fail
R0.5G0.8	4.340836	14.14791	17.36334	18.97106	20.57878
R1G0	22.51037	40.9751	Fail	Fail	Fail
R1G0.4	5.588585	15.57669	18.90606	21.40309	22.82996
R1G0.8	2.49584	8.818636	10.81198	11.47587	12.47255
R2G0	11.26025	33.4079	Fail	Fail	Fail
R2G0.4	1.781377	10.93117	16.68016	18.21862	19.83806
R2G0.8	1.06383	2.70793	6.479691	10.34816	11.89555

In the absence of glutaraldehyde, scaffolds were destroyed within a month

### Comparison of fabricated scaffolds

Scaffold porosity in all types of samples was capable of handling the size of chondrocytes and their biological function. Considering the average tensile strength and Young’s modulus of the human trachea i.e. 4.08×10^2^±0.96×10^2^ kPa[25] and 2.5-7.7 MPa[35], respectively, R1G0.8, R2G0.8, and R2G0.4 were chosen as the most mechanically compatible samples with human trachea (Table 3). However, the R2G0.4 was excluded due to poor degradability rate[36]. Hence, biocompatibility and cell characterization were performed on R1G0.8 and R2G0.8 samples to choose an optimized scaffold for tracheal tissue engineering.

**Table 3:** Characterizations of selected scaffolds according to mechanical propertieas and degradation rate. Young’s modolus of theses scaffolds were in the range of the Young’s modolus oh the human tracheal cartilage i.e. 8.76 to 16.92 MPa

Scaffold	Strength (kPa)	Young’s modulus (MPa)	Weight loss after 70 days (%)
R2G0.4	265.74±25.12	9.9±2.08	19.83±4.54
R2G0.8	354.8±33.7	12.2±2.4	11.89 ±2.07
R1G0.8	263.5±18.34	7.5±1.97	12.47±3.87

### *In vitro* cell culture

#### MTT assay

**Fig. 6:**
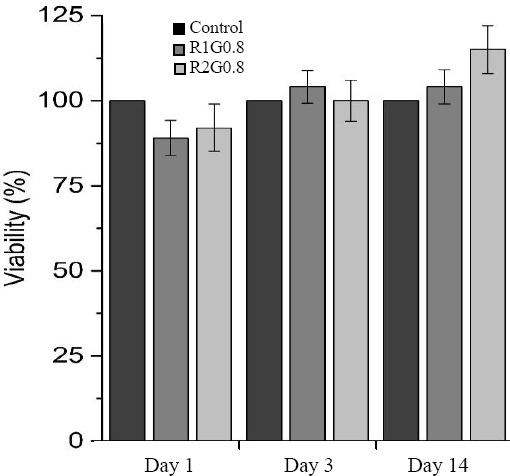
The viability of cell seeded scaffolds at 1, 3, and 14 days. All samples have a proliferation rate more than control group. There was no toxicity in the concentration of 0.8 wt% of GA.

To determine the toxicity of GA concentration, an extraction process was performed based on ISO 10993-12[29]. Figure 6 shows the viability of the cells exposed to the scaffolds, which were cross-linked by 0.8 wt% of GA at different freezing rates including 1 and 2°c/ min, on days 1, 3, and 14.

Results indicated no major cytotoxic secretion for all samples. There were also no significant differences in cell viability on days 1 and 3 in comparison with the control group on the same day of culture.

As the previous studies documented, chitosan is a potentially favorable biomaterial for the attachment and proliferation of chondrocyte and plays an important role in preserving the phenotype and capacity to synthesize collagen II and GAG[38,39]. In the case of the proliferation rate, both R2G0.8 and R1G0.8 had the proliferation rate higher than the control group. In addition, cell viability was significantly improved on day 14 for sample R2G0.8 compared to the control group (*P*<0.05).

#### SEM observation of the cells

**Fig. 7:**
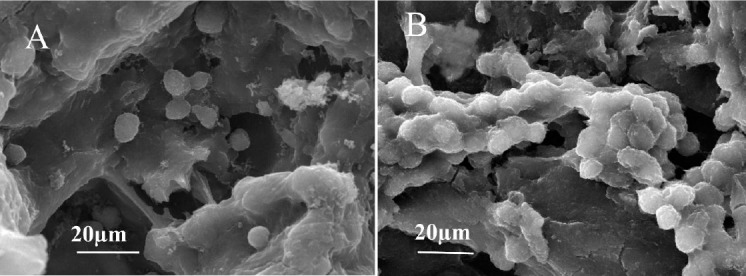
Scanning electron microscopy image of cell attachment on day 21. Chondrocyte culture on R1G0.8 (A), and R2G0.8 (B). Chondrocytes are embedded in the scaffolds. Spherical shape of the cells is in accordance with chondrocyte phenotypes.

The morphology of the cells seeded on scaffolds on days 14 and 21 were observed using SEM (Fig. 7). The cells were attached to the scaffolds well, and the cell morphology was similar to that of chondrocytes, especially on day 21.

#### Determination of glycosaminoglycans

**Fig. 8:**
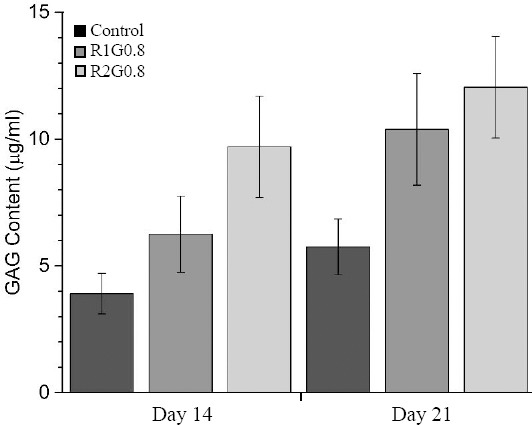
Glycosaminoglycan (GAG) content of control group and cell-seeded scaffolds on days 14 and 21. GAG release was significantly higher in R2G0.8 compared to R1G0.8 on days 14 (P<0.05). However, the same result was not achieved on day 21.

The values of released GAG for different samples are shown in Figure 8. GAG content was increased on days 14 and 21 in R2G0.8 in comparison with R1G0.8 and control group. There was a significant difference between R1G0.8 and R2G0.8 compared to control group (*P*<0.05). GAG release was also significantly higher in R2G0.8 compared to R1G0.8 on days 14 (*P*<0.05) but statistical analysis demonstrated no significant difference in GAG content of R2G0.8 and R1G0.8 on day 21.

## DISCUSSION

In this study, chitosan silk scaffolds were fabricated for tracheal tissue engineering using freeze-casting technique. Three different consolidation rates along with three concentrations of GA were applied to make a variety of composites in terms of mechanical properties and porosity. Based on the tensile strength, Young’s modulus, degradation rate, and pore size, two appropriate scaffolds were chosen for *in vitro* cell culture. The results of 21-day cell culture indicated that both 1 and 2°C/min of the freezing rates and 0.8 wt% GA would make a non-toxic, porous structure having tensile strength and elastic modulus compatible with the human trachea. Furthermore, cell adhesion and DMMB assay confirmed the chondrogenic character of the scaffolds.

GA is a widely used crosslinking agent due to being easily accessible and affordable[40]. At the other extreme, GA, as a free agent, is toxic because of crosslinking the intracellular proteins[41], although toxicity is dose-dependent, and up to 8% GA has been shown to be non-cytotoxic[42]. Moreover, the use of GA at low concentration allows obtaining stable materials by creating new bonds in composite without any toxic effect[43,44]. In this regard, Wu *et al*.[45] utilized GA in different concentrations as a crosslinker with no report of cytotoxicity. In our study, no destructive effects on chondrocytes were observed to be caused by the concentration of 0.8 wt% GA similar to the data reported by Komura and coworkers[46] based on crosslinking gelatin sponges with 0.16 wt% GA. GA concentration affects mechanical characteristics, swelling ratio, and degradation, which are significant features of an optimized scaffold. As it can be seen in the FTIR spectra (Fig. 1), GA created new bonds in the structure, which resulted in the interaction between polymers’ molecules. It increases the molecular weight leading to enhanced mechanical properties and reduced degradation rate[47].

In the present study, by adding 0.4 wt% and 0.8 wt% GA, the strength of scaffolds (Fig. 4A) in all three freezing rates was elevated from 172.06 kPa (mean tensile strength of R0.5G0 , R1G0, and R2G0) to 210 kPa (mean tensile strength of R0.5G0.4 , R1G0.4, and R2G0.4) and 267.9 kPa (mean tensile strength of R0.5G0.8, R1G0.8, and R2G0.8), respectively. Average Young’s modulus was increased by 45.95% and 84.76% through an increment of GA concentration as well (Fig. 4B).

In terms of swelling behavior, both silk fibroin and chitosan are hydrophilic biomaterials[48]. The results of PBS uptake of the scaffolds showed the moderately high swelling capacity of the structures (Fig. 5). The swelling behavior can be diminished by rising the GA amount[40]. Indeed, 0.4 wt% and 0.8 wt% GA decreased the swelling ratio almost 3% and 7%, respectively. The swelling behavior and degradation rate were more affected by GA concentration rather than freezing rate. To be more precise, at the rate of 0.5°C/min, the average PBS absorption was 29.54% and varied weakly with respect to the changes in freezing rate. In the consolidation rate of 1 and 2°C/min, the PBS absorption was decreased to 27.7% and 24.75%, respectively. From a biological point of view, the fluid content of hyaline cartilage, as the most important part of the trachea, was about 80% of the extracellular matrix (ECM)[48,50]. Therefore, the scaffolds with high absorption capacity may mimic the ECM texture for cartilage tissue engineering.

Considering degradation results, although R2G0.4 and R1G.08 had similar tensile strength values, their degradation rates were different. While a fast degradation was observed in R2G0.4 samples (about 18% degradation in two months), those samples with 0.8 wt% GA had approximately 10% degradation within the same period. Meanwhile, in the absence of GA, disintegration arose within one month.

Along with GA concentration and the presence of silk fibroin in the structures, freeze-casting parameters (temperature, solute concentration, direction, and freezing rate) influenced the PBS absorption, mechanical behavior, and degradability of the scaffolds. The main mechanism of the freeze-casting method is that the particles are leaded by water molecules and packed between ice crystals. The freezing rate is a significant factor affecting ice nucleation, crystal growth, and consequently the size and morphology of the pores[49-51]. It also allows particles to be rearranged after dispersing; therefore, the lower freezing rate would create a lower porosity scaffold and denser pack due to more time for the rearrangement of particles[50]. Additionally, the lamellar structure obtained by freeze casting is under the influence of freezing rate. Indeed, the higher freezing rate, the thinner are the lamellar structures[51]. Low temperature (-196°C) of liquid nitrogen also results in small ice crystals compared to higher temperatures[52]. Moreover, an increase in solidification rate leads to the smaller size of pores, which in turn lowers the PBS uptake. Accordingly, tensile stress and Young’s modulus will be increased.

In terms of pore size, SEM images (Fig. 2) denoted a homogenous morphology of the pores in both directions. Pore sizes were larger in the parallel than perpendicular direction, in the ranges of 372.06±34.26×83.41±22.21 µm to 135.31±25.74 ×37.83±13.03 µm that was suitable for chondrocyte culture[34]. The typical pore size must allow cells to migrate through the structure and be able to transport biomolecules and growth factors. Scaffold heterogeneity could lead to a non-uniform cell attachment and affect the ability of the cells to produce a homogenous ECM with proper Young’s modulus[40]. Hence, among various scaffold fabrication techniques, freeze casting, as an inexpensive method, and at a constant freezing rate can be employed to create more uniform porous scaffolds for trachea engineering applications. Since tracheal scaffolds are required to be laterally rigid and longitudinally flexible[1], the growth of ice crystals can be orientated in one direction by controlling the direction of freezing and the pores can be formed in ideal conditions. Other methods including electro-spinning and solvent casting have the limitations such as lack of reproducibility, creation of non-uniform pores, and the toxicity risk of the remained solvent [53]. However, the ease of application, low cost, and accessibility of freeze drying make it a good candidate for scaffold fabrication.

Several investigations have confirmed the capability of chitosan silk fibroin composites for cartilage regeneration, which is a key element in providing mechanical integrity in the tracheal tissue. Previously, based on these materials, a composite scaffold with the tensile stress and elastic modulus of 1.88×10^2^ kPa and 2.47×10^2^ kPa have been fabricated to be used in trachea tissue engineering[25]. With respect to the crosslinking agent as well as frigidity of -196°C, we here fabricated a composite with the mechanical characteristics closer to the human trachea, while other structural and biological requirements were met. All three tracheal components, including trachealis muscle, connective tissue, and cartilage demonstrated the well-known “stiffening” behavior. The mean value of elastic modulus of human tracheal cartilage has been estimated to be from 8.76 to 16.92 MPa[37].

SEM images showed that the scaffolds maintained high cell attachment over the time. Furthermore, GAG content was increased in samples particularly in R2G0.8, which showed higher Young’s modulus. As a matter of fact, the structural configuration in terms of porosity and mechanical behavior acted as a suitable conductor of chondrocytes. The presence of GAG-like structure supported neo-chondrogenesis and facilitated chondrocytes proliferation on the scaffold surface *in vitro*.

Appropriate dimensions of patient’s trachea are accessible by synthetic scaffolds in comparison with acellular trachea as a biologic scaffold. However, the biocompatibility and biogegradability of these types of scaffolds are the most restrictive factors. In this regard, natural materilas are suitable candidates but their biomechanical insufficiency is the main weakpoint that can be addressed using composite structures along with crosslinking agents.

The ability to control the freezing rate and to apply crosslinking agents allows optimizing pore morphology over a physiologically meaningful condition and serves as a unique method for tailoring particular tissue requirements. Cartilage tissue regeneration is the target point of the tracheal engineering. Both R1G0.8 and R2G0.8 facilitated the rabbit chondrocyte adherance and prolifration and provided an excellent porosity for cell attachment at the optimized mechanical environment.
